# Recurrent Stimulation of Natural Killer Cell Clones with K562 Expressing Membrane-Bound Interleukin-21 Affects Their Phenotype, Interferon-γ Production, and Lifespan

**DOI:** 10.3390/ijms20020443

**Published:** 2019-01-21

**Authors:** Maria A. Streltsova, Sofya A. Erokhina, Leonid M. Kanevskiy, Maria V. Grechikhina, Polina A. Kobyzeva, Dean A. Lee, William G. Telford, Alexander M. Sapozhnikov, Elena I. Kovalenko

**Affiliations:** 1 Shemyakin and Ovchinnikov Institute of Bioorganic Chemistry, Russian Academy of Sciences, ul. Miklukho-Maklaya, Moscow 117997, Russia; mstreltsova@mail.ru (M.A.S.); sonya.erokhina@gmail.com (S.A.E.); lmkanevskiy@gmail.com (L.M.K.); marygrec@mail.ru (M.V.G.); polina-kobyzev@yandex.ru (P.A.K.); amsap@mx.ibch.ru (A.M.S.); 2 Center for Childhood Cancer and Blood Disorders, The Research Institute, Nationwide Children’s Hospital, Columbus, OH 43205, USA; dean.lee@nationwidechildrens.org; 3 Experimental Transplantation and Immunology Branch, National Cancer Institute, National Institutes of Health, Bethesda, MD 20892, USA; telfordw@mail.nih.gov

**Keywords:** NK cell clones, IL-2, K562-mbIL21, membrane-bound IL-21

## Abstract

A pattern of natural killer cell (NK cell) heterogeneity determines proliferative and functional responses to activating stimuli in individuals. Obtaining the progeny of a single cell by cloning the original population is one of the ways to study NK cell heterogeneity. In this work, we sorted single cells into a plate and stimulated them via interleukin (IL)-2 and gene-modified K562 feeder cells that expressed membrane-bound IL-21 (K562-mbIL21), which led to a generation of phenotypically confirmed and functionally active NK cell clones. Next, we applied two models of clone cultivation, which differently affected their phenotype, lifespan, and functional activity. The first model, which included weekly restimulation of clones with K562-mbIL21 and IL-2, resulted in the generation of relatively short-lived (5–7 weeks) clones of highly activated NK cells. Levels of human leukocyte antigen class II molecule—DR isotype (HLA-DR) expression in the expanded NK cells correlated strongly with interferon-γ (IFN-γ) production. The second model, in which NK cells were restimulated weekly with IL-2 alone and once on the sixth week with K562-mbIL21 and IL-2, produced long-lived clones (8–14 weeks) that expanded up to 10^7^ cells with a lower ability to produce IFN-γ. Our method is applicable for studying variability in phenotype, proliferative, and functional activity of certain NK cell progeny in response to the stimulation, which may help in selecting NK cells best suited for clinical use.

## 1. Introduction

The phenotype of natural killer cells (NK cells) substantially changes during their differentiation and activation, forming heterogeneous subpopulations with various expression of surface receptors, effector molecules, and signal proteins, as well as different functional activity [1,2]. The mechanisms of NK cell differentiation are not completely clear. During this process, due to certain epigenetic changes, NK cells lose the expression of the NKG2A/CD94 receptor and begin to express inhibitory killer-cell immunoglobulin-like receptors (KIRs), as well as cluster of differentiation (CD)57, the cell maturation marker. Occasionally, and often in association with cytomegalovirus infection, highly differentiated NK cells form subsets of adaptive-like cells that intensively express activating KIR and NKG2C receptors [3,4]. Moreover, the background of phenotypic alterations, proliferative, and functional activity of NK cell changes includes cytotoxicity and the production of cytokines. Generation and analysis of the individual NK cell progeny helps to characterize more precisely the differentiation and activation processes on the single-cell level and to study the functional features of NK cells at different maturation stages that may have advantages in the developing of approaches for the expansion of NK cells for clinical applications. Previously, clonally expanded NK cells were used to study distribution of CD94/NKG2A and CD94/NKG2C heterodimers [5]; changes in CD94/NKG2 receptor expression depended on the functional activity of NK cells [6]. It was shown that heterogeneity in NK cell cytotoxic activity was related to various expression levels of different activating and inhibitory receptors in NK cells. Therefore, the role of KIR receptors in HLA-uncoordinated hematopoietic stem cell transplantation was studied in a number of works using NK cell clones [7,8]. The level of anti-tumor NK cell cytotoxicity was shown to be dependent on KIR repertoire acquired during NK cell differentiation. Further, functional tests utilizing NK cell clones may help in defining cytotoxicity in NK cells for different tumor variants [8].

Several techniques were designed to produce NK cell clones. One of the first methods was based on the use of limiting the dilution and cultivation of cells in a medium supplemented with irradiated (EBV)-transformed B cells or irradiated allogeneic lymphocytes as feeder cells [9,10]. In other studies, the method of co-cultivation of NK cells with dendritic cells was used to obtain NK cell clonal expansion [11,12]. In the year 2000, a protocol was published describing stable production of NK cell clones by the limiting dilution method using RPMI-8866 feeder cells [13]. However, since the method of limiting dilutions does not guarantee obtaining progeny of a single cell, some researchers began to apply sorting technique in the “single cell” mode to obtain cell clones [14,15,16,17]. The stimulation platform applied in the current study, with the use of gene-modified K562 feeder cells expressing membrane-bound interleukin (IL)-21 (K562-mbIL21) in combination with IL-2, was previously shown to induce stable proliferation and significant expansion of NK cells [18,19]. IL-2 stimulates proliferation and differentiation of NK cells and increases their functional activity [20,21]. According to various data, IL-21 co-stimulates proliferation, promotes maturation of NK cells, enhances their functional activity, induces the production of interferon-γ (IFN-γ) [22], and increases cytotoxicity [20,23]. It is shown that IL-21-stimulated NK cells not only have a direct antitumor effect, but also affect naive and activated T cells, causing their migration and differentiation [24]. Along with IL-2, IL-21 has been described as a promising cytokine that enhances the antitumor properties of NK cells ex vivo [25]. Both of these cytokines may be useful for obtaining NK cells for immunotherapeutic applications by enhancing innate antitumor response of NK cells. The first results from clinical trials (phase I) that used mbIL21-expanded haploidentical NK cells for the treatment of leukemia patients after transplantation of allogeneic hemapoietic stem cells demonstrated low toxicity and promising therapeutic effects [26]. Because of the significant heterogeneity, a final NK cell population obtained after in vitro stimulation and expansion does not always satisfy the necessary requirements concerning subset composition and anti-tumor activity. NK cell cloning allows to select cells with desirable properties for further adoptive cancer immunotherapy. Another problem of using in vitro expanded NK cells, which should be accumulated in large numbers for adoptive transfer, is their decreased cytotoxic activity after storage in a frozen state. Obtaining NK cells that do not lose their functional activity after defrosting would allow them to accumulate in advance and use for treatment as soon as they are needed. Several groups have searched for optimal cryopreservation strategies, applicable for expanded NK cells [27,28,29]. In the current study, we tested the permanence of markers expression and functional activity of NK cell clones, cultivated according to model 2 (described above), that suggested freezing and defrosting the cells after prolonged storage.

The method of NK cell cloning proposed in this study is based on the stimulation of sorting called the “single cell” mode, where NK cells with gene-modified K562 feeder cells express membrane-bound IL-21 (K562-mbIL21) in combination with IL-2. We developed two models that further cultivated the obtained NK cell clones, which resulted in a different phenotype and lifetime of clones. Model 1 implies weekly addition of the feeder cells, paired together with a partial substitution of the IL-2-supported medium. This cultivation model produced NK cell clones with a shorter clone lifespan (5–7 weeks), activating phenotype and higher IFN-γ production, compared to model 2, where the addition of K562-mbIL21 feeder cells occurred once during incubation, at week six. On the other hand, the cultivation of model 2 allowed us to increase the clone lifespan up to 14 weeks. These clones retained natural cytotoxicity, but most of them had lower CD16 and HLA-DR expression levels, especially when compared to clones cultivated according to model 1. Above all, we demonstrated that cryopreservation of well-proliferating NK cell clones cultivated using model 2 had almost no effect on their phenotype and functional activity. 

## 2. Results

### 2.1. NK Cell Cloning Conditions

**Figure 1:**
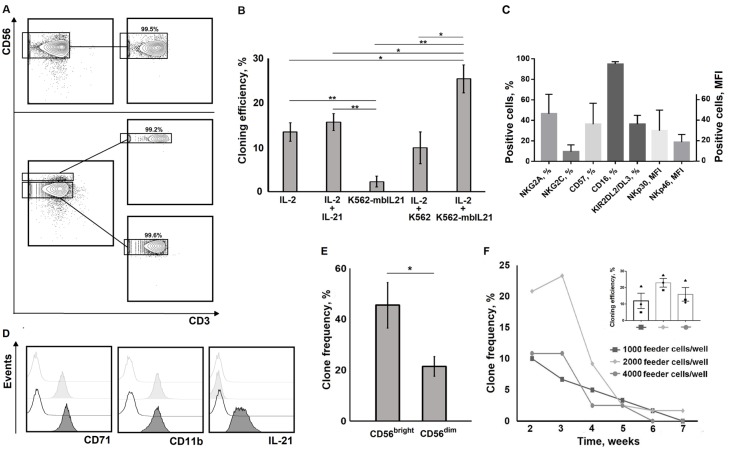
Selection and optimization of the natural killer cells (NK cells) cloning method. (**A**) Gaiting strategy for sorting individual NK cells and purity of resulted cell fractions (1—whole NK cell population; 2— cluster of differentiation (CD) 56^bright^ and CD56^dim^ subsets). Staining of magnetically separated NK cell fraction was performed with CD56 and CD3. (**B**) The efficiency of clone formation (cloning efficiency) using different stimuli. Mean ± SE of *n* independent experiments is presented (*n* = 3 for IL-2; *n* = 4 for IL-2 + IL-21; *n* = 3 for gene-modified K562 feeder cells expressing membrane-bound IL-21 (K562-mbIL21); *n* = 3 for interleukin (IL)-2 + K562; *n* = 5 for IL-2 + K562-mbIL21). (**C**) Phenotypic analysis of ex vivo NK cells before sorting. Mean ± SD of NK cell samples of eight individuals is shown. (**D**) Comparative phenotypic characterization of K562 (light grey) and K562-mbIL21 (dark grey) cells. CD71, CD11b, and IL-21 staining and isotype controls are presented. (**E**) CD56^bright^ NK cells generate more clones than CD56^dim^. Data of four clone collections are presented in each column. (**F**) Selection of the number of K562-mbIL21 feeder cells for obtaining human NK cell clones. Cloning efficiency was calculated as clone frequency at the indicated week, when the greatest number of clones was detected in a collection. Data of three independent experiments are presented in the columns. NK cells of three donors (indicated by different symbols) were independently cloned. Significant differences are shown by asterisks as * *p* < 0.05; ** *p* < 0.01.

We cloned the NK cell cloning via FACS sorting the NK cells into 96-well plates (Figure 1). The advantage of this method over the method of limiting dilutions is that all wells most likely contain one NK cell per well. Several combinations of cytokines and feeder cells, including K562 cells and modified K562-mbIL21 cells, were tested for NK cell cloning in order to maximize the efficiency of clone generation (Figure 1B,D). The cloning efficiency was calculated in each collection as clone frequency at the week, when the greatest number of clones was counted. Stimulation of NK cells with IL-2 alone, IL-2 + IL-21 (10 ng/mL), or IL-2 + unmodified K562 cells (10^4^ cells/mL) resulted in cell proliferation in only 10–15% of the wells containing single NK cells, with minor differences between stimuli combinations (Figure 1B). Still, IL-2 was essential for NK cell clone generation.

Thus, IL-21 or unmodified K562 had no additional impact on clone frequency, whereas IL-2 was required for NK cell clone generation. NK cells stimulated with modified K562-mbIL21 feeder cells alone demonstrated very low clone generation efficiency (Figure 1B). The clones, obtained with IL-2 alone, IL-2 + IL-21, or IL-2 + unmodified K562, lived no more than 4–5 weeks. However, when NK cells were cultivated in the presence of IL-2 in combination with K562-mbIL21, the efficiency of the clone generation increased significantly, reaching 30% or more in certain experiments. Moreover, using this method, we were able to obtain long-lived clones of certain NK cells (up to 14 weeks). Some variations in cloning efficiency were found for NK cells isolated from different donors. We did not find a clear association of the clone generation frequency with expression levels of NK cell receptors, including NKG2A, NKG2C, CD16, KIR2DL2/DL3, NKp30, and NKp46, which varied in ex vivo NK cells within intervals typical for healthy individuals (Figure 1C). Proportion of CD56^bright^ subset was on average 4.87% (SD = 2.46) in initial NK cell fractions. Notably, when CD56^bright^ and CD56^dim^ NK cell subsets gated during cell sorting and cloned separately, the frequency of clones was higher in the fraction of CD56^bright^ cells, compared to CD56^dim^ NK cells (Figure 1E). CD56^dim^ cells also responded to IL-2, but formed less clones. 

In order to select optimal conditions for clone generation, we compared the efficiency of clone formation using several feeder cell concentrations per well (Figure 1F). The efficiency was the greatest at 2 × 10^3^ feeder cells per well and the survival of the obtained NK cell clones in this case was more prolonged, especially when compared to other stimulation conditions (Figure 1F). Therefore, the optimal conditions for NK cell clone generation appeared to be 100 U/mL of IL-2 and 2 × 10^3^ K562-mbIL21 cells per well (Figure 1).

### 2.2. Restimulation Frequency Affects NK Cell Clones Lifespan, Phenotype, and Functional State

**Figure 2:**
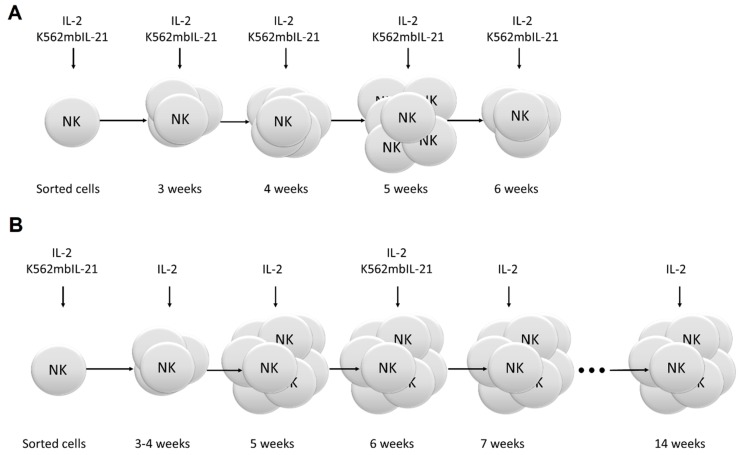
Schemes of NK cell clone cultivation procedures. (**A**) Model 1—weekly addition of feeder cells, starting from the third week. (**B**) Model 2—single addition of feeder cells at week six.

We studied the influence of restimulation frequency on NK cell clone formation and survival, as the effect of feeder cells may depend on the time and duration of their addition [30]. In model 1, K562-mbIL21 feeder cells combined with IL-2 were added to NK cells every week after clonal expansion was registered (usually at week three). In model 2, feeder cells were added to NK cell clones once during cultivation and once at week six; IL-2 was added weekly. In both models, initial cloning conditions were the same (100 U/mL IL-2 and 2 × 10^3^ K562-mbIL21 cells per well) (Figure 2).

Clones cultivated using model 1 generally had a shorter life-span than clones cultivated using model 2. In the three collections of clones obtained from different donors with model 1, the life-span of all clones did not exceed 5–7 weeks. Average clone survival in week five was around 40% (Table 1). In contrast, when model 2 was used, the lifespan of some individual NK cell clones ranged between 8 and 14 weeks, or sometimes more. In total, 550 clones in six collections were obtained using model 2. Among them, 86 (15.6%) were long-lived clones with the lifespan lasting for eight weeks or more. Of these clones, 10 (11.6%) showed the greatest lifespan (12–14 weeks). After five weeks of cultivation, the total number of cells in clonal cell cultures was obtained using model 1, reaching values ranging from 5 × 10^4^ to 3 × 10^6^ cells in one clone (Table 1). Within clones cultivated according to model 2, the variability in expansion rates was much higher. In seven weeks, 20% of the proliferating clones produced more than 5 × 10^6^ cells. In three individual clones cultured using model 2, the total cell number reached 1–2 × 10^7^ cells. 

**Figure 3:**
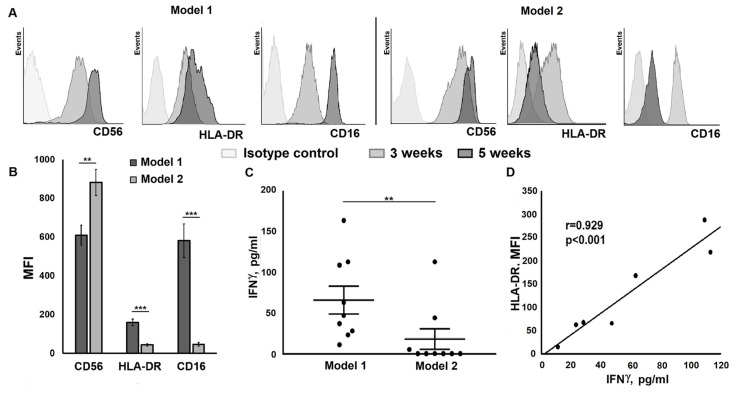
Expression of surface markers and interferon-γ (IFN-γ) production by NK cell clones depends on the cultivation model. (**A**) Dynamics of surface markers expression in NK cell clones cultivated according to model 1 and model 2. Measurements were carried out at weeks three and five of cultivation. (**B**) Differences in the expression of CD56, human leukocyte antigen-DR isotype (HLA-DR), and CD16 in NK cell clones cultivated using models 1 and 2 (measurements were carried out at week 5). (**C**) Comparison of the level of IFN-γ in supernatants of clones cultivated according to models 1 and 2 (from 22 to 67 independent clones were analyzed in each type of measurement). (**D**) Correlation between HLA-DR expression level and IFN-γ production in clones cultivated using model 1. (**C**,**D**) Measurements were carried out at week four of clone cultivation. Significant differences are shown by asterisks as ** *p* < 0.01; *** *p* < 0.005.

We then analyzed the phenotype of the obtained clones by measuring the expression of different NK cell markers. All of the clones were CD56-positive and CD3-negative. The level of CD56 expression increased during cultivation, regardless of the model of cultivation (Figure 3A). 

The expression level of HLA-DR, measured after five weeks of cultivation, was higher in clones restimulated weekly with IL-2/K562-mbIL21 (model 1), when compared to clones with one feeder cell restimulated at week six, as well as and weekly restimulation with IL-2 alone (model 2) (Figure 3B). Normally, a part of human peripheral blood NK cells express HLA-DR and its function is still unknown, but it is considered to be an activation and/or proliferation marker [31]. We observed that repeated additions of the IL-21-expressing feeder cells during cultivation gradually increased the expression of HLA-DR in NK cell clones. In clones cultivated according to model 2, high expression of HLA-DR was observed three weeks after culture initiation, but decreased later even in well-proliferating clones (Figure 3A).

Many of the clones produced IFN-γ in response to IL-2. The intensity of production varied significantly between clones. Statistical analysis revealed significant differences in the production of IFN-γ between clones cultivated using models 1 and 2 (Figure 3C). All analyzed clones cultivated using model 1 produced IFN-γ. In contrast, clones cultivated using model 2 demonstrated IFN-γ production in less than 50% of the analyzed clones. Importantly, we found a strong correlation between IFN-γ production intensity and HLA-DR expression levels in NK cell clones cultivated according to model 1 (Figure 3D). Thus, weekly restimulation with IL-2 + K562-mbIL21 feeder cells, but not with IL-2 alone induced coordinated increase in HLA-DR expression and IFN-γ production. The increase of HLA-DR expression was observed in some but not all proliferating NK cell clones, suggesting that HLA-DR expression was somehow connected with IFN-γ production but was not a reliable proliferation marker for NK cell clones.

In addition to HLA-DR, CD16 expression also increased during clone cultivation in model 1. In clones cultivated using model 2, CD16 expression level measured at week five was lower than at week three (Figure 3A). On average, the expression level of CD16 in clones cultivated according to model 1 at week five was significantly higher than clones cultivated according to model 2 at the same time. Interestingly, despite the similar averaged dynamics of HLA-DR and CD16 expression in the clones cultivated by the same method, we found no significant correlation between CD16 and HLA-DR expression intensity in individual clones.

### 2.3. Changes in Certain Clone Characteristics are Associated with the Addition of Feeder Cells

**Figure 4:**
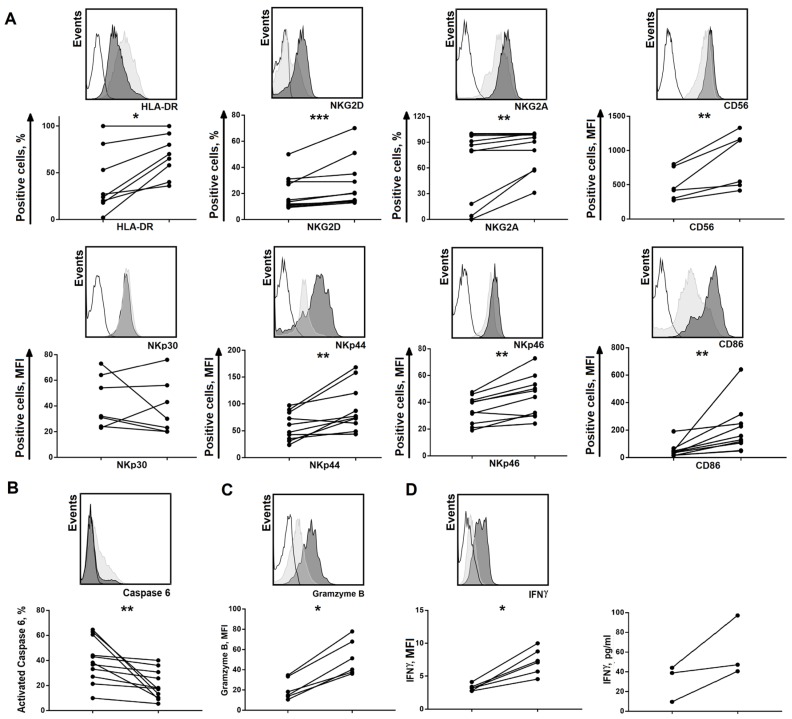
Phenotypic and functional analysis of NK cell clones stimulated using IL-2 with or without K562-mbIL21 feeder cells. (**A**) Phenotypic analysis of clones stimulated using IL-2 with or without K562-mbIL21 feeder cells for two weeks. The clones were divided into two equal parts after six weeks of cultivation using model 2 and then one part was restimulated with feeder K562-mbIL21 cells. Histograms show representative staining of an individual clone cultivated with (dark grey) or without K562-mbIL21 (light grey). The changes in surface markers cultured clones without (left) and with the addition of feeder cells (right). (**B**) Natural cytotoxicity of clones stimulated using IL-2 without (light grey; left) and with the addition of feeder cells (dark grey; right) measured during week four of cultivation. (**C**) Granzyme B intracellular levels in clones cultivated without (light grey; left) and in the presence of feeder cells (dark grey; right). (**D**) IFN-γ production via clones stimulated using IL-2 without (light grey; left) or with (dark grey; right) K562-mbIL21 feeder cells. Intracellular IFN-γ levels and IFN-γ concentrations in culture supernatants are presented. Wilcoxon signed rang test was used for the determination of statistical differences. Significant differences are shown by asterisks as * *p* < 0.05; ** *p* < 0.01; *** *p* < 0.005.

To determine the effect of feeder cells on clone phenotype, several clones cultivated using model 2 were divided into two equal parts at week 6. Then these clones were cultivated for two weeks in the presence of IL-2 (100 U/mL) and feeder cells were added weekly only to one half of the cells of each clone; feeder cells were added weekly. Then, surface marker expression was assessed in these cells (Figure 4A). 

Clone cells cultured with the addition of feeder cells were more activated compared to the cells stimulated with IL-2 alone, as seen by the increase in the expression levels of HLA-DR, CD86, and NKp44 (the activation markers). The surface levels of CD56, activating receptors NKp46 and NKG2D, and the inhibitory receptor, NKG2A, also increased. Thus, contact interactions between NK cells and feeder cells lead to additional activation of clonal populations.

In further experiments, functional activity of clones obtained with cultivation models 1 and 2 was compared. For this, the selected clones were divided into two parts at week two of cultivation. One part was cultivated according to model 1 with weekly restimulation using feeder cells; the second part was conducted according to model 2 and without the addition of feeder cells. The levels of natural cytotoxicity, intracellular granzyme B, and IFN-γ production were evaluated. A statistically significant decrease in the level of natural cytotoxicity was found in clones cultivated with the addition of K562-mbIL21 feeder cells, especially when compared to the clones solely stimulated with IL-2 (Figure 4B). At the same time, clones restimulated with feeder cells had increased intracellular granzyme B levels (Figure 4C). To determine the potency to produce IFN-γ, the cells were transferred to the medium without interleukins for 24 h and then restimulated with IL-12 + IL-15 for 18 h. We found that contact interactions with feeder cells during cultivation increased the level of IFN-γ production (Figure 4D), which confirms the data in Figure 3C.

### 2.4. Long-Lived NK Cell Clones Obtained Using Model 2 were Capable of Natural Cytotoxicity

**Figure 5:**
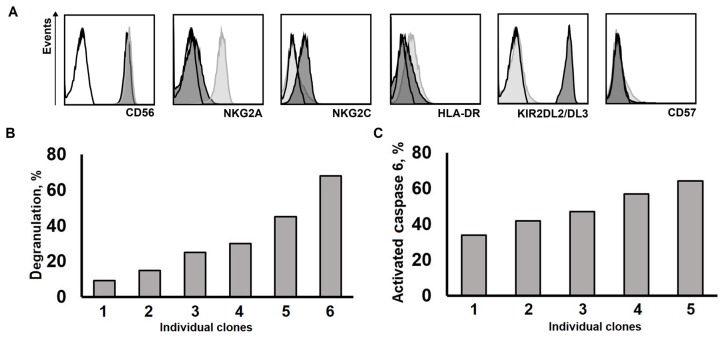
Analysis of the phenotype and functional activity of long-lived NK cell clones. (**A**) Phenotypic analysis of NK cells clones performed at week 10 of culturing. Two phenotypic patterns (light grey and dark grey) are shown. (**B**) Degranulation of the clones, measured by the level CD107a expression on the cell surface after incubation in the presence of K562 target cells at week 14 of cultivation. (**C**) The efficacy of natural cytotoxicity of the clones, measured by caspase-6 activation in K562 target cells at week 14 of cultivation.

Since only clones cultivated according to model 2 were alive for more than eight weeks, we analyzed phenotype and cytotoxic capacity of several well proliferating clones of this series late in their culturing. We found two main phenotypic patterns of NK cell clones cultivated for 10 or more weeks (Figure 5A). The first pattern included a high expression level of NKG2A, the absence or presence of KIR2DL2/DL3 expression, and the absence of NKG2C on the cell surface. The second pattern was characterized by low NKG2A level, KIR2DL2/DL3 positivity, and by the presence of NKG2C expression (Figure 5A). This pattern seemed to be rare; it was observed only in one clone analyzed in this series. All clones were positive for CD56 and most of them expressed HLA-DR at significant levels, but were mostly negative for CD57.

We demonstrated that even after 10–14 weeks of cultivating NK cell clones, their growth within model 2 retained the ability to degranulate, showing a significant cytotoxic potential against standard K562 target cells, although the levels of natural cytotoxicity varied greatly between clones (Figure 5A,B).

### 2.5. Freezing NK Cell Clones Retains Their Functional Potential

To study the effect of freezing on the phenotype and functional status of the clones, some of the long-lived NK cell clones cultivated according to model 2 were frozen and then defrosted after a year. It was found that freezing clones at the concentration below 1.5 million/mL led to an unstable recovery of NK cells from the frozen state, which negatively affected the lifetime of the clone after defrosting. Clones with different proliferative activity were used for freezing. The level of proliferation was assessed via the increment in the number of cells per week of incubation, which was calculated according to the formula N_2_/N_1_, where N_1_ is the initial number of cells per mL of medium and N_2_ is the number of cells per ml of medium after one week of incubation. Clones with the highest chance of survival after defrosting had an increment iindex of two or higher. The lifetime of clones after defrosting was at least three weeks. As a result, optimal conditions for the freezing, which provided stable proliferation of clones after defrosting, were selected.

**Figure 6:**
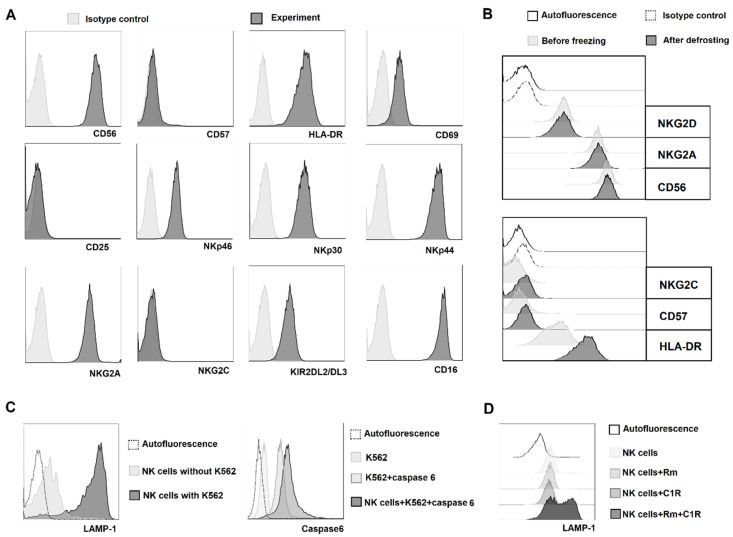
Analysis of the phenotypic and functional characteristics of defrosted NK cell clones, generated using model 2. After defrosting, clones were cultivated in NK cell medium with 100 U/mL IL-2 for one week. The figure shows the data of one representative clone. (**A**) Analysis of several surface marker expression in clones, measured by flow cytometry after defrosting. (**B**) Comparison of surface expression of surface receptors and markers before freezing (light gray) and after defrosting (dark gray). (**C**) Analysis of natural cytotoxicity in defrosted NK cell clones by evaluating CD107a expression (left) and the level of activated caspase 6 in K562 target cells (right). (**D**) Analysis of antibody-dependent cell cytotoxicity in defrosted NK cell clones. CIR cell line (B cell lymphoma) was used as target with humanized anti-CD20 antibody Rituximab.

Defrosted clones were cultivated for a week in complete medium with IL-2 (100 U/mL) and then were functionally and phenotypically characterized (Figure 6A). 

All clones were CD56^+^CD57^−^NKG2A^+^, which is characteristic for less differentiated NK cells. After defrosting, surface expression of the main markers in the clones was similar to the expression measured before freezing (Figure 6B). Furthermore, defrosted clones were functionally active in both natural (Figure 6C) and antibody-dependent cellular cytotoxicity tests (Figure 6D).

## 3. Discussion

NK cell clones are commonly used for studies of NK cell differentiation [16,32], phenotypic, and functional characteristics of individual NK cells [5,6], as well as for the selection of NK cells with increased anti-tumor activity [7,8]. Development of an effective method for generation and expansion of NK cell clones for NK cell-based immunotherapy can help avoid the problem of heterogeneity of functional and phenotypic characteristics of donor NK cells. 

A variety of stimulation options can be applied to activate and expand NK cells [18,33,34]. Some of them can be used to obtain NK cell clones [13,16]. Most of the stimulation approaches are based on combinations of cytokines acting cooperatively with NK cells. Previously, a combination of IL-2 and K562mbIL-21 had been shown to induce intensive proliferation of NK cells [18,19]; this approach can be successfully used to obtain cells for adoptive immunotherapy [26]. This method of stimulation was chosen for the generation of NK cell clones in the current work. 

Because of the significant heterogeneity of initial NK cell fraction, the final NK cell population obtained after in vitro stimulation and expansion may not always satisfy the necessary requirements for subset composition and anti-tumor activity. NK cell cloning allows for the selection of cells with desirable properties for further adoptive cancer immunotherapy. At the moment, there are a few protocols describing a method of stable production of NK cell clones. The method used in the current study allows for the obtainment of homogeneous cell populations with higher accuracy than the limiting dilution method described in the Cella and Colonna protocol [13]. The acquisition of long-lived clones has been described in work of Carr et al. [17]; they reported that the obtained clones had been cultivated for 8–16 weeks. However, their technique requires weekly addition of two types of feeder cells—allogeneic peripheral blood mononuclear cells (PBMC) and Epstein–Barr virus (EBV)-transformed B lymphoblastoid cell line—into wells with growing clones. The production of allogeneic PBMC is associated with additional difficulties. In contrast to this method, we propose a model for the stable production of long-lived clones, which requires only two-fold introduction of one type of feeder cells. Feeder cells based on the cell line are more accessible and easier to use. Since feeder cells are eliminated within a week after their addition, it is possible to obtain a pure culture of NK cells, which does not require special purification from feeder cells, for example, on the ficoll gradient.

IL-2 and IL-21 receptors contain a common IL-2 receptor (IL-2R) γ-chain, which explains the similarity in the effect of these cytokines on NK cells, although there are also a number of differences in signaling pathways [35]. The balance of signaling components underlies the complex regulation of NK cell activation and functioning mediated by these cytokines. In our stimulation system, IL-21 was presented to NK cells on the surface of K562 cells. These feeder cells expressed the 4-1BBL ligand, whose receptor is expressed by NK cells [36]. Clones obtained with such combined stimulation lived longer than clones obtained with IL-2 alone. Empirically, we defined the optimal dose of feeder cells (2 × 10^3^ cells per well of a 96-well round-bottom plate), which was sufficient to stimulate NK cell expansion (Figure 1F). The results agree with data from earlier works describing co-stimulatory effects of IL-21 on IL-2-induced proliferation of NK cells [30,37]. 

The highest cloning frequency was registered in the CD56^bright^ subset of NK cells. These cells are thought to respond better to IL-2, expressing a high affinity receptor for this cytokine. The longest-living clones (up to 14 weeks or more) were obtained using model 2, in which feeder cells expressing IL-21 were added to NK cells at week one and then again at week six. More frequent addition of feeder cells led to a shorter lifespan of the clones, usually no more than 5–7 weeks. Several studies have already demonstrated that the effects of IL-21 on the expansion rate and phenotypic characteristics depend on the dose and, most interestingly, on the timing and duration of exposure. For instance, the addition of IL-21 during the first week of cultivation caused an increase in the muber of NK cells at the moment of culture initiation [30]; whereas, during repeating stimulation with IL-21 in combination with IL-2, IL-21-mediated restriction of proliferation was observed [30,38]. Continuous exposure to IL-21 can make the proapoptotic effect of this cytokine more pronounced [22]. Multiple additions of feeder cells can also lead to the excessive degranulation of NK cells, which exhausts their intracellular resources and adversely affects their survival. The most significant expansion of NK cells (10^11^-fold after six weeks) described in the literature was observed within a single initial stimulation with IL-21 [39]. In our work, more than 2 × 10^7^ cells per clone were obtained in certain clones, specifically when a membrane-bound IL-21 was added twice: at the beginning of cultivation and at week six. Single restimulation of NK cells by the feeder cells also resulted in a significant increase in the cell number up to week six, but without restimulation, the proliferation rate of NK cells after six weeks of cultivation slowed, and the death of clones was registered (data not shown).

Stimulation with IL-2 and/or IL-21 results in an increase of HLA-DR expression on the surface of NK cells [40,41,42]. Thus, HLA-DR has been proposed as a marker of NK cell activation. Furthermore, IL-21 in both its soluble and membrane-bound form augments the production of IFN-γ in NK cells [30,43]. In this and earlier studies by our group, HLA-DR expression was detected in NK cell clones that were obtained using a combined stimulation with IL-2/K562-mbIL21 [23]. A higher level of HLA-DR expression has been observed in clones grown with a weekly addition of IL-2/K562-mbIL-21, compared to clones solely restimulated with IL-2 (Figure 3C), indicating the significant role of IL-21-expressing feeder cells in increasing the expression of HLA-DR. CD16 surface level was also higher in clones cultivated by model 1 than in model 2. Interestingly, HLA-DR level, but not CD16 level, correlated positively with the intensity of IFN-γ production. In clones that had cultivated with the single addition of feeder cells at week six, along with the weekly addition of IL-2 (model 2), IFN-γ production significantly decreased throughout cultivation, alongside with the decrease in HLA-DR and CD16 expression (Figure 3). A slight decrease in CD16 expression was observed in another study, when IL-21 was applied at the initial stage of stimulation [30,41]. Significant changes in CD16 expression and intensity was observed in both of the cultivation models, which indicates that CD16 should not be considered a differentiation marker in vitro. 

The lower level of proliferative activity and short lifetime of clones cultivated using model 1 makes this method less attractive for immunotherapy. Nonetheless, a prolonged lifespan of clones cultivated using model 2 goes along with lower surface expression of CD16 and the production of IFN-γ, which is also unfavorable for immunotherapeutic NK cell applications. We have tried to find a way to increase the functional activity of clones cultivated using model 2 during their growth. It is known that restimulation of NK cells via cytokines leads to an increase in their functional activity. In particular, cytokine-induced NK memory cells can be obtained by such a method [44]. We hypothesized that feeder cells can impact the NK cell functions in a similar way. We have investigated whether NK cell clones can be activated at a later period of clonal expansion with the restimulation of feeder cells. The twice-repeated addition of feeder cells significantly increased expression levels of activation-associated markers in clones (Figure 4). The possibility to increase the expression level of surface molecules at various time periods makes it possible to change the functionality of clones cultivated using method 2, simultaneously taking advantage of more intense proliferation and a longer lifespan of such clones, which can be useful for personalized therapy. In addition, long-lived clones cultivated using model 2 retained natural cytotoxicity at a later time period and demonstrated the ability to kill targets even after 14 weeks of cultivation. All this makes model 2 a promising approach for a generation of NK cells with desired properties. Interestingly, within these long-lived clones, we have found a clone demonstrating some phenotypic characteristics that is typical for memory-like NK cells: it was positive for NKG2C and negative for NKG2A. However, in contrast to classical phenotype of adaptive NK cells [3,4], it was almost negative for CD57. In-depth analysis of phenotypic, proliferative, and functional characteristics of such clones may be the subject of future study. With regard to model 1, the weekly restimulation of clones by feeder cells may lead to a decrease in natural cytotoxicity, apparently because of exhausting of cytolytic potential of NK cells (Figure 4B). Intriguingly, these clones had higher intracellular levels of granzyme B, one of the main mediators of granule-mediated NK cell cytotoxicity (Figure 4C). It can also be assumed that the functional activity is somehow connected to the proliferative potential of the clone.

The use of the present cloning technique is to obtain a homogeneous population of NK cells for personalized immunotherapy, which is possible only if the cells retain their characteristics after freezing/defrosting. This is a potential limitation for the use of NK cells in immunotherapy, because unlike T cells, they are very sensitive to the process of freezing and thawing, which leads to loss of their functional activity. Our results showed that even prolonged freezing (within one year) does not affect the phenotype and does not reduce the functional activity of NK cell clones. Clones, recovered after cryopreservation, demonstrated high expression levels of a number of surface markers and good proliferative capacity (Figure 6). They also demonstrated a high level of natural and antibody-dependent cytotoxic activity. Thus, freezing/defrosting of clones, generated with IL2 + K562mbIL-21, does not affect their phenotypic and functional characteristics. 

## 4. Material and Methods

### 4.1. Cell Lines

K562 cells obtained from the American Type Culture Collection (ATCC, Manassas, VA, USA) and modified K562-mbIL21 cells also expressing CD64, CD86, CD137L (4-1BBL), and truncated CD19 produced earlier [18] were cultivated using a complete RPMI-1640 medium, i.e., RPMI-1640 supplemented with 10% fetal calf serum (FCS) (HyClone, Logan, UT, USA), 2 mM of L-glutamine (PanEco, Moscow, RF), and an antibiotic-antimycotic solution (Millipore-Sigma, St. Louis, MO, USA), at the concentration of 2–6 × 10^5^ cells/mL. Before using for NK cell stimulation, K562-mbIL21 cells were irradiated with γ-radiation (100 Gy) and immediately frozen in FCS containing 10% DMSO (Sigma-Aldrich, St. Louis, MO, USA) and stored at −135 or −150 °C. The C1R cell line was obtained from ATCC and cultivated using a complete RPMI-1640 medium at the concentration of 2–6 × 10^5^ cells/mL.

### 4.2. NK Cell Isolation

Blood samples were taken from healthy volunteers of different age and sex. All participants gave their informed consent prior to the study in accordance with the recommendations of the local ethics committee of Pirogov Russian National Research Medical University (protocol #169 of the ethics committee meeting, 20.11.2017). NK cells were isolated by ficoll density gradient centrifugation of peripheral blood samples, followed by negative magnetic separation using a human NK cell isolation kit (Miltenyi Biotec, Bergish Gladbach, Germany) according to the manufacturer’s protocol. 

### 4.3. Preparation of Plates with Feeder Cells for Cell Sorting

The complete NK cell medium consisted of 80% DMEM medium (PanEco, Moscow, RF), 20% xx-vivo medium (Lonza, Basel, Switzerland), or AIM-V medium (Gibco-Thermo Fisher Scientific, Waltham, MA, USA) supplemented with 100 units/mL of recombinant IL-2 (Sigma-Aldrich, St. Louis, MO, USA). This medium was mixed with 10^4^ of irradiated K562-mbIL21 feeder cells per mL and filled into 60 central wells of a 96-well plate (200 µL). The marginal wells were filled with RPMI-1640 medium to minimize evaporation from the central wells during cultivation of sorted NK cells.

### 4.4. Fluorescence-Activated Single Cell Sorting and Generation of NK Cell Clones

Purified NK cells were labeled with mouse anti-human monoclonal antibodies (mAbs) CD3-PE-Cy7 (Beckman Coulter, Brea, CA, USA, clone UCHT1) and CD56-Brilliant Violet 421 (Sony Biotechnology Inc., San Jose, CA, USA, clone HCD56). CD3^−^CD56^+^ cells were sorted into 96-well round-bottom plates containing feeder cells suspended in complete NK cell medium, one cell per well in the “single cell” mode. Cell sorting was performed with FACSVantage DiVa machine (BD Biosciences, San Jose, CA, USA) equipped with 405, 488, and 643 nm lasers, as well as an appropriate set of detectors and filters. The plates were then put into a CO_2_-incubator (5% CO_2_, 37 °C) for two weeks. After eight weeks of incubation, clones were transferred to a new 96-well round-bottom plate.

### 4.5. Cultivation of NK Cell Clones

#### 4.5.1. Model 1

Every week beginning from week three, 100 µL of the medium was substituted with the same volume of suspension containing complete NK cell medium with 100 unit/mL of recombinant IL-2 (Sigma-Aldrich, St. Louis, MO, USA) and 10^4^/mL of irradiated K562-mbIL21 feeder cells (Figure 2A).

#### 4.5.2. Model 2

Every week beginning from week three or four, 100 µL of the medium was substituted with the same volume of fresh complete NK cell medium containing 100 unit/mL of recombinant IL-2 (Sigma-Aldrich). 10^4^/mL of irradiated K562-mbIL21 feeder cells were added once after six weeks of clone cultivation (Figure 2B).

### 4.6. Estimation of NK Cell Clone Generation Frequency and Lifespan

Clone generation frequency (cloning efficiency) was calculated according to the formula Fc = Fa/Fb * 100% (Fc—clone generation frequency, Fa—number of wells with clones, Fb—total number of occupied wells) in a week, when the maximal number of clones was registered. Depending on the donor, the maximum number of clones could be obtained at different time points, from two to four weeks of incubation. The percentage of surviving clones was calculated as the ratio of the number of clones detected at a certain week to the maximum number of clones detected throughout the whole experiment (usually at week two, three, our four, depending on the collection).

### 4.7. Surface Fluorescent Immunostaining and Flow Cytometry

To study the surface marker expression in NK cells, the following mAbs were used: CD3-PE-Cy7 (clone UCHT1), CD56-APC (clone N901), CD56-PE (clone N901 (HLDA6)), HLA-DR-FITC (clone B8.12.2), from Beckman Coulter; CD56-Brilliant Violet 421 (clone HCD56), CD16-PE (clone 3g8), from eBioscience, an Affymetrix Company, Santa Clara, CA, USA; CD57-PE (clone TB01), NKp46-FITC (clone 9E2), NKp30-PE (clone P30-15), NKp44-PE (clone 44.189), CD107a-PE-Cy5 (clone H4A3), NKG2D-PE (clone 1D11), CD69-PE (clone FN50), CD11b-PE-Cy7 (clone M1/70), from Sony Biotechnology; CD57-FITC (clone TB03), CD57-APC (clone TB03), anti-KIR2DL2/DL3-PE (clone DX27), anti-IFNγ-FITC (clone REA600), from Miltenyi Biotech; anti-NKG2A-PE (clone 131411), anti-NKG2C-PE (clone 134591), anti-NKG2A-PE (clone 131411), R&D Systems, Minneapolis, MN, USA; CD25-FITC (clone M-A251), CD71-PE (clone M-A712), from BD Biosciences, San Jose, CA, USA, CD86-PE (clone IT2), anti-IL-21-PE (clone 3A3-N2), anti-Granzyme B-Alexa Fluor 647 (clone GB11), from BioLegend, San Diego, CA, USA.

Surface fluorescent immunostaining was performed for 30 min on ice in PBA staining buffer (PBS containing 0.5% BSA (bovine serum albumin) (Serva, Heidelberg, Germany) and 0.01% sodium azide (AMRESCO, Inc. (VWR International, LLC), Aurora, CO, USA)). After washing twice with PBA, samples were analyzed using FACSCalibur flow cytometer (BD Biosciences, USA), equipped with 488 and 640 nm lasers. At least 30,000 events in lymphocyte gate for total NK cells and 5000 events for NK cell clones were recorded. Acquired data were analyzed using FlowJo program version 7.6 (FlowJo LLC, Ashland, OR, USA) and Flowing Software version 2.5.1 (PerttuTerho, Turku Centre for Biotechnology, Finland).

### 4.8. Natural Cytotoxicity Evaluation

The K562 cell line was used as a target for analysis. The experiments were carried out at a 1:1 ratio of NK cells:targets. Killing effectiveness was measured by two methods.

#### 4.8.1. Registration of Caspase-6 Activity in Target Cells

Analysis of cytotoxicity based on the percentage of target cells with active caspase-6 was performed using a CyToxiLux kit (CTL602, Oncoimmunin, Gaithersburg, MD, USA). Briefly, K562 cells were pre-stained with vital TFL4 dye in RPMI-1640 medium and then washed. NK cells and K562 cells were mixed and centrifuged at 240 g for five min. After decantation of the supernatant, 50 μL of caspase-6 substrate (CS) was added, cells were pelleted at 240 g for 30 s and then incubated for 30 min at 37 °C, 5% CO_2_. After washing with PBA, samples were analyzed with FACSCalibur flow cytometer, percentage of TFL^+^CS^+^ K562 cells was evaluated.

#### 4.8.2. Registration of NK Cell Degranulation by CD107a Expression

The experiments were carried out according to the procedure described earlier [23,45]. Briefly, NK cells were mixed with K562 cells in the RPMI-1640 medium with CD107a-PE-Cy5 antibody (eBioscience, USA, clone H4A3) and 10 μg/mL of brefeldin A (Sigma, USA). Cells were precipitated at 240 g for 30 s and then incubated for 2.5 h at 37 °C, 5% CO_2_. Analysis was performed on the FACSCalibur flow cytometer; the percentage of CD107a^+^ NK cells was evaluated.

### 4.9. Antibody-Dependent Cytotoxicity Evaluation

C1R target cells were added to NK cells at a 1:3 ratio (target:effector) in RPMI-1640 medium containing brefeldin A, 10 μg/mL, CD107a-PE-Cy5 mAb, 0.5 μg/100 μL, and Rituximab (CD20 mAb, Roche Holding, Basel, Switzerland), 2.5 μg/mL. Cells were precipitated at 240 g for 30 s and incubated for 2.5 h at 37 °C, 5% CO_2_. Analysis of NK cell degranulation was performed on a FACSCalibur flow cytometer.

### 4.10. Analysis of IFN-γ Production Assessment 

#### 4.10.1. ELISA

IFN-γ secretion potential of clones was analyzed at week five of clone cultivation. Prior to analysis, NK cell clones were stimulated with cytokines using two methods. According to the first method, 10^5^ of clone cells were incubated in fresh complete NK cell medium with 100 U/mL of IL-2 overnight. According to the second method, 10^5^ of clone cells were incubated in fresh complete NK cell medium without any stimuli for 24 h and then restimulated with 10 ng/mL of IL-12 and 10 ng/mL of IL-15 for 16 h. Then, NK cell clone supernatants were collected and the level of secreted IFN-γ was analyzed as described earlier [46] using ELISA kit (Vector-Best, Moscow, RF). 

#### 4.10.2. Intracellular Staining

2 × 10^5^ cells were incubated with fresh complete medium without IL-2 overnight and restimulated with 10 ng/mL IL-12 и 10 ng/mL IL-15 within 18 h. Brefeldin A (10 ng/mL, Sigma) was added to the cells for last 4 h of incubation. Then, cells were stained with CD56-PE, fixed, and permeabilizated using BD Cytofix/Cytoperm™ kit (BD Biosciences), according to the manufacturer’s instructions. For intracellular staining samples, cells were incubated with anti-IFNγ antibody in BD Perm/Wash™ buffer for 30 min on ice, then washed and analyzed using flow cytometry. 

### 4.11. Intracellular Granzyme B Evaluation

Here, 2 × 10^5^ cells were fixed and permeabilizated using a BD Cytofix/Cytoperm™ kit (BD Biosciences). Then, samples were incubated with anti-Granzyme B antibody in BD Perm/Wash™ buffer for 30 min on ice, washed, and analyzed.

### 4.12. Statistical Analysis

For determining the statistical significance of results, a student’s *t*-test was used for data with normal distribution. A Mann-Whitney U-test and a Wilcoxon signed rang test were used for data having non-normally distribution. Finally, *p*-values of < 0.05 were considered significant.

## 5. Conclusions

To conclude, in the current study, we have developed two models for NK cell clonal expansion that produced cells with different phenotype and functional activity. Weekly restimulation with IL-2 and K562-mbIL-21 feeder cells (model 1) resulted in higher expression of HLA-DR, CD16, and granzyme B and high production of IFN-γ in NK cell clones, i.e., it led to the development of clones with activated phenotypes. Such clones demonstrated shorter lifespans and lower levels of proliferative activity. On the contrary, the use of cultivation in model 2 (weekly restimulation of clones with IL-2 in the absence of feeder cells) resulted in a generation of long-lived highly proliferating clones with a lower level of IFN-γ secretion, but still retaining abilities for natural cytotoxicity. Depending on the objectives of the study, a choice should be made in favor of a particular model of NK cell cloning and cultivating. The method of human NK cell clone generation using K562mbIL-21 feeder cells can be considered as a way to obtain homogenous NK cell populations, which might be more effective for adoptive immunotherapeutic treatment. The possibility to freeze such NK cell clones without the loss of functional activity allows for the creation of a bank of NK cells for personalized immunotherapy. 

## Figures and Tables

**Table 1:** Survival rates and expansion levels of NK cell clones obtained in model 1 and model 2. Total cell numbers were counted in randomly selected well proliferating clones.

Model	Survival (Mean ± SD)		Total Cell Number in Well Proliferation Clones (Mean ± SD)		
	Week 5	Week 7	Week 5 (19 Clones)	Week 7 (25 Clones)	Week 12 (10 Clones)
Model 1 (3 collections)	42% ± 5%		1.2 × 10^6^ ± 1.14 × 10^6^		
Model 2 (6 collections)		30% ± 18%		2.8 × 10^6^ ± 4.0 × 10^6^	3.9 × 10^6^ ± 5.84 × 10^6^

## Abbreviations

IFN-γ	interferon-γ
IL	interleukin
K562-mbIL21	gene-modified K562 cells expressing membrane-bound IL-21
NK cells	natural killer cells
PBMC	peripheral blood mononuclear cells
